# From measuring noise toward integrated single-cell biology

**DOI:** 10.3389/fgene.2014.00408

**Published:** 2014-11-19

**Authors:** Pawel Paszek

**Affiliations:** Faculty of Life Science, University of ManchesterManchester, UK

**Keywords:** single-cell analysis, single-cell genomics, cellular heterogeneity, biological noise, measuring noise

A single cell is inherently noisy. This noise is observed as a variability or heterogeneity between individual cells' responses in an isogenic population, and emerges from fundamental physical process governing state of the cell over time. In practice, states of two seemingly identical cells may be different in the same environment; and in fact the behavior of the population average my not correspond to any of the individual cells. Recent decades brought a technological breakthrough in many areas in our ability to measure and interpret cellular heterogeneity, including live-cell imaging (Spiller et al., 2010) and genome-wide epigenetic and expression analyses (in particular next generation sequencing) (Chattopadhyay et al., 2014). The emerging picture is that the cellular noise is not a nuisance, but a ubiquitous functional trait that could perhaps be therapeutically exploited. Here we discuss relevant technological advances as well as postulate the need for more quantitative and integrated temporal single cell biology approaches to study cellular heterogeneity.

## Quantifying cellular noise

Recent technological advances in single-cell bioassays transformed our ability to measure cellular noise and heterogeneity. They vary in the number of parameters that can be simultaneously monitored, the spatio-temporal resolution, and the ability to provide quantitative understanding (Table 1).

**Table 1:** **Review of single-cell biology approaches**.

	**Content (number of targets)**	**Temporal resolution**	**Quantification**	**Genetic engineering**
Live cell fluorescence imaging	<10	Yes (very high)	Yes (absolute)	Yes
Live cell luminescence	<3	Yes (very high)	Yes (relative)	Yes
Intracellular dyes for imaging	<10	Yes (very high)	No (relative)	No
RNA fish	<5 (~1 for live cell)	No (yes for live cell)	Yes (absolute)	No/Yes for live cell
Flow cytometry	<20	No	Low (relative)	No
Mass cytometry	<40	No	Low (relative)	No
Antibody capture assays	<5	Some	Low (relative)	No
Digital RT-qPCR	<100	No	Yes (relative)	No
Genomics	~10,000	No	Yes (relative)	No

“Seeing is believing” and thus the fundamental breakthrough to measure cellular noise has been the use of genetically encoded fluorescent or luminescent probes for live cell microscopy (Spiller et al., 2010). This is by far the *most temporarily and spatially resolved, and quantitative approach* (Locke and Elowitz, 2009), which over last few decades provided a step change in our ability to visualize heterogeneity, for example noisy gene expression (Molina et al., 2013) or all-or-none cellular sensing mechanisms (Tay et al., 2010). The key applications involve genetically engineered systems for expression of protein fusions for monitoring of cellular dynamics, or promoter-driven reporters for analysis of transcriptional responses. In fact, the combination of both, for example simultaneous monitoring of RNA (by labeling individual transcripts, see below) and protein level in real-time provides more statistical power for dynamical correlation studies of gene expression noise (Larson et al., 2013). More advanced imaging methods such as Förster Resonance Energy Transfer (FRET) and Fluorescence Recovery After Photobleaching (FRAP) also exist. They allow detailed measurements of molecular interactions and intra-cellular movement, and ultimately absolute quantifications of number of reacting molecules, for example via Fluorescence Correlation Spectroscopy (FCS) (Spiller et al., 2010). The advance of live-cell imaging have been aided with the development of tracking algorithms for analysis of time-laps movies, an essential part of the data quantification pipeline (Shen et al., 2006; Zambrano et al., 2014) as well as microfluidic systems for manipulations of individual cells on the microscope (Yin and Marshall, 2012). This together enabled automatic high-throughput analyses of hundreds of cells at a time, removing the analysis bottleneck. The imaging approach however is quite limited by the number of probes that can be simultaneously resolved and applications monitoring more that three processes are still rare (Gerlich et al., 2001). Better more spectrally resolved fluorophores are requited, although combination of fluorescent and luminescent probes allows further multi-parametrization. At the same time, engineering of model systems is not trivial. Viral (Payne, 2007) and Bacterial Artificial Chromosome (Gong et al., 2010) systems are commonly used to stably introduce transgenes into cells, with the latter providing more contextual expression, especially for reporter (promoter-dependent) systems. However, more physiological approaches such as CRISPR also emerged allowing labeling endogenous genes in their full epigenetic context (Jinek et al., 2012). Moving from cell lines into animal models proves to be even a more challenging task, simply because of the Mendellian genetics affecting transgene transmissions and thus limiting ability to multiplex different probes.

A notable part of the imaging approach has been the development of RNA fluorescence *in situ* hybridization (FISH) methods. They rely on targeting transcripts with fluorescently labeled oligonucleotide probes in fixed cells (Raj et al., 2008). RNA FISH allows spatial visualization and *absolute quantification* of *individual transcripts* in cells *without the need for genetic engineering*, and thus contextual expression can be measured. As with live-cell microscopy, physical properties of fluorescent probes only allow simultaneous measurements of limited number of probes (Singer et al., 2014). The *static* nature of RNA FISH measurement is it largest limitation; however a substantial effort has been made toward visualization of transcripts in living cells. A key development arrived with a MS2 system based on a RNA-binding protein (derived from bacteriophage MS2) that is capable of targeting transcripts, when tagged with a specific recognition motif (Bertrand et al., 1998). Subsequently MS2 system has been successfully transferred into an animal model with a development of transgenic mouse expressing MS2 probe for β-actin RNA (Lionnet et al., 2011). While gaining the temporal resolution, live-cell RNA FISH requires genetic engineering. Methods for labeling of endogenous transcripts in living cell also have been introduced, for example using human RNA-binding protein PUMILIO1, which recognizes the target RNA sequence rather than the secondary structure as in case of MS2 protein (Ozawa et al., 2007). However, multiplexing for live cell RNA imaging is still limited by the lack of well-characterized RNA-binding motifs, and sensitivity required for detection of relative small number of target molecules.

## Toward high-content measurements: targeting proteins

The natural drive in the field has been toward multi-parameter measurements that are beyond capability of live-cell imaging. These initially stemmed from the antibody-based recognition systems, which were historically used to phenotype cellular populations. After years of development, current antibody based fluorescent-activated flow cytometry (FACS) systems now enable measurements of up to 20 parameters simultaneously (Chattopadhyay et al., 2006). This has been possible due to developments of vast array of chemically or biologically derived dyes as well as advances in laser technology and equipment (Bendall et al., 2012). An interesting approach is the combination of flow cytometry with microscopy by ImageStream cytometers, which in addition of standard antibody-based phenotyping provides additional parameters by enabling visualization of protein localization or cell morphology (Rao et al., 2012). The most recent innovation includes mass cytometry, theoretically capable of measuring up to 100 parameters (practically up to 40), which uses purified isotopes rather than fluorescent dyes to label antibodies, and therefore can be resolved by mass spectroscopy (Bjornson et al., 2013). Unfortunately, single-cell methods for traditional label-free mass spectrometry are not fully developed yet, only allowing analysis of selected, usually very highly abundant proteins or metabolites (Rubakhin et al., 2011). Most recent developments in the antibody-based approaches involve miniaturization of assays into microfluidic capture devices (Lu et al., 2013). These essentially enable measuring proteins secreted by individual cells in sophisticated micro-plate systems, and thus facilitate *temporal analyses*. In general, the antibody-based methods are capable of providing a quite rich single-cell data, with some *degree of quantification*, however there still relatively limited to much fewer than 100 simultaneous measurements.

## The single-cell genomic revolution

A major step change in the field occurred when the well-established “omics” technology, in particular next generation sequencing was applied to single cells. This, over few recent years transformed our ability to measure cellular heterogeneity from monitoring few sometimes arbitrarily selected markers into unbiased genome-wide analyses (Eberwine et al., 2014). In addition to providing ability to find and phenotype “rare” cells, single-cell genomic data provided more statistical power for association studies. Of the “omics” methods, single-cell transcriptomics (scRNA-seq) is the most advanced and widely used in the field. In just few years, scRNA-seq enabled characterizing transcriptional landscapes of immune (Shalek et al., 2014), cancer (Ramskold et al., 2012) and embryonic (Islam et al., 2011) cells, among others. The method enabled high content decomposition of cellular heterogeneity within a healthy (Jaitin et al., 2014) or cancer (Patel et al., 2014) tissues, as well as demonstrated single-cell splicing events and random mono-allelic gene expression patterns (Deng et al., 2014). What follows now is the development of methods for studying the epigenome regulation led by establishment of single-cell bisulfite sequencing for measurements of DNA methylation (Smallwood et al., 2014) or nucleosome mapping (Small et al., 2014). While whole genome single-cell DNA amplifications are possible, there is still a sensitivity gap for studying DNA-protein interactions or histone modifications due to additional purification steps required (Gilfillan et al., 2012).

Single-cell genomics is still very much an emerging field and thus many questions remain about reproducibility of data and normalization standards required (Wu et al., 2014). A simple fact that a minimal amount of material can be collected from a cell (~10 pg of DNA and ~20 pg of RNA) implies the need for ample amplification before the measurement can be taken. The amplification process naturally introduces bias toward more highly abundant molecules, affecting the dynamical range of the measurement and specifically detection of low-level expressing targets. Many normalization and quality-control protocols are used to improve quantitative aspects of this analysis. The most rigorous controls involve combination of cell and molecular barcoding (so called unique molecular identifiers, UMI); with allows labeling of individual transcripts prior to amplification and thus detecting individual transcript molecules (Jaitin et al., 2014). Other methods include external RNA spike-in controls for amplification bias, as well as RNA FISH or digital RT-qPCR for transcript distribution normalizations. The latter is capable of multiplexing up to 96 transcripts in 96 single cells when combined with microfluidics, for example C1 system (Wu et al., 2014), and thus it is a single-cell method with its own merit. The depth of sequencing required for single cell analyses is also debatable. Initial studies used depths of the order of standard population level experiments (~10 mln reads), however recent studies utilizing UMI barcoding suggest that as low as 50 k reads per cell is sufficient to measure up to 10 k unique transcript molecules per cell (Jaitin et al., 2014). This allows massive parallelization with thousands of cell analyzed simultaneously and thus reducing the cost. Despite this, single-cell sequencing methods require further optimization as current estimations suggest that only a small fraction of transcripts in a cell can be harvested and measured.

## Toward more integrated temporal analyses

The picture emerging from the single-cell methods is that of unprecedented, previously unobservable levels of noise and heterogeneity in single cells. This noise emerges via all-on-nothing (Tay et al., 2010) or graded (Warmflash et al., 2012) transcription factor activation in fluctuating environments, the downstream complex dynamics involving pulsatile and oscillatory patterns (Paszek et al., 2010; Levine et al., 2013), the cross-talk with other intracellular signaling systems and cell-to-cell communication, which ultimately drives heterogeneous gene and protein expression patterns at the genome wide-scale (Shalek et al., 2014). However, despite the recent revolution in single-cell biology very few methods exist that is capable of integrating different measurements. These are needed for the association studies between different levels of cellular organization, and ultimately the understanding of how the noise propagates in individual cells and tissues.

However, is the static measurement going to be sufficient for studying associations? One could expect that integrated genome-wide studies would enable correlating for example epigenetic, transcriptomic, and proteomic heterogeneity. However, existing analyses already suggested that this correlation might be in fact smaller than expected (Taniguchi et al., 2010). More importantly, the ultimate task of resolving how noise and heterogeneity contributes to disease, which is often associated with heterogeneous and complex genetic traits may be even more challenging.

In the inference of dynamical systems the *statistical power* and *causality* comes from the *quantitative and temporal* rather than static measurements under informative *perturbation*. This is best exemplified by a number of theoretical analyses and inference of cellular noise (Bowsher and Swain, 2014). In the near future no one expects a temporal resolution in genomic data, therefore the breakthrough has to come from elsewhere [although in some biological systems, pseudo-temporal ordering of cells undergoing differentiation programmes can be obtained; Trapnell et al., 2014]. One approach is the integration of existing temporal approaches, e.g., live-cell imaging with genomic and proteomic end-point assays, for example using microfluidic systems or micro dissection techniques for cell isolation. However, it is the integration of different temporal approaches that perhaps can provide a step-change in the field. While simultaneous RNA and protein visualization has already been achieved (Larson et al., 2013), much richer dataset are required. One avenue to provide those temporally resolved data is integrating imaging and antibody-based (e.g., capture) assays. In addition, more precise ways to perturb cell are also required, for example such as optogenetic (Deisseroth, 2011), and nanowire approaches (Shalek et al., 2010). These will allow dissecting contributions of complex intracellular networks that eventually control the genome-wide heterogeneity.

Biological systems are inherently complex, and a noisy cell is their common denominator. The single cell biology approach is ideally suited to resolve this noise and heterogeneity with further developments of more integrated and quantitative approaches.

### Conflict of interest statement

The author declares that the research was conducted in the absence of any commercial or financial relationships that could be construed as a potential conflict of interest.
